# Resuscitation with macromolecular superoxide dismutase/catalase mimetic polynitroxylated PEGylated hemoglobin offers neuroprotection in guinea pigs after traumatic brain injury combined with hemorrhage shock

**DOI:** 10.1186/s12868-020-00571-7

**Published:** 2020-05-13

**Authors:** Soichiro Seno, Jun Wang, Suyi Cao, Manda Saraswati, Sharon Park, Jan Simoni, Li Ma, Bohdan Soltys, Carleton J. C. Hsia, Raymond C. Koehler, Courtney L. Robertson

**Affiliations:** 1 Department of Anesthesiology and Critical Care Medicine, Johns Hopkins University, 600 North Wolfe Street, Blalock 1404, Baltimore, MD USA; 2 Division of Traumatology, Research Institute, National Defense Medical College, Saitama, Japan; 3 AntiRadical Therapeutics, Sioux Falls, SD USA; 4 Department of Physics, Georgia Southern University, Statesboro, GA USA

**Keywords:** Carbon monoxide, Controlled cortical impact, Guinea pig, Hemoglobin-based oxygen carrier, Hippocampus, Neuroprotection

## Abstract

**Background:**

Polynitroxylated PEGylated hemoglobin (PNPH, aka SanFlow) possesses superoxide dismutase/catalase mimetic activities that may directly protect the brain from oxidative stress. Stabilization of PNPH with bound carbon monoxide prevents methemoglobin formation during storage and permits it to serve as a carbon monoxide donor. We determined whether small volume transfusion of hyperoncotic PNPH is neuroprotective in a polytrauma model of traumatic brain injury (TBI) plus hemorrhagic shock. Guinea pigs were used because, like humans, they do not synthesize their own ascorbic acid, which is important in reducing methemoglobin.

**Results:**

TBI was produced by controlled cortical impact and was followed by 20 mL/kg hemorrhage to a mean arterial pressure (MAP) of 40 mmHg. At 90 min, animals were resuscitated with 20 mL/kg lactated Ringer’s solution or 10 mL/kg PNPH. Resuscitation with PNPH significantly augmented the early recovery of MAP after hemorrhagic shock by 10–18 mmHg; whole blood methemoglobin was only 1% higher and carboxyhemoglobin was 2% higher. At 9 days of recovery, unbiased stereology analysis revealed that, compared to animals resuscitated with lactated Ringer’s solution, those treated with PNPH had significantly more viable neurons in the hippocampus CA1 + 2 region (59 ± 10% versus 87 ± 18% of sham and naïve mean value) and in the dentate gyrus (70 ± 21% versus 96 ± 24%; n = 12 per group).

**Conclusion:**

PNPH may serve as a small-volume resuscitation fluid for polytrauma involving TBI and hemorrhagic shock. The neuroprotection afforded by PNPH seen in other species was sustained in a species without endogenous ascorbic acid synthesis, thereby supporting potential translatability for human use.

## Background

Moderate and severe traumatic brain injuries (TBIs) typically produce an immediate mechanical injury followed by delayed secondary injury to surrounding tissue. Furthermore, some TBI victims suffer multi-trauma accompanied by significant hemorrhage. The resulting arterial hypotension can exacerbate neuronal injury. Thus, rapidly restoring and sustaining mean arterial pressure (MAP) in multi-trauma victims with TBI and hemorrhagic shock (HS) is of critical importance for initial treatment [1]. Protocols for HS employ rapid infusion of crystalloid or colloid fluids in the field, followed by blood transfusions as needed in a hospital setting. However, definitive treatment with blood, platelets, coagulation therapy, and surgery can be delayed by many hours, especially in remote locations or in situations with large military and civilian casualties [1]. In these situations, an ideal resuscitation fluid should have multiple targets. It should not only serve as a resuscitation fluid that restores and sustains MAP in victims with TBI and HS, it should also protect neurons and the entire neurovascular unit from secondary injury cascades, such as those resulting from reactive oxygen species. Furthermore, fluids that require smaller volumes for successful resuscitation can have logistical advantages in these scenarios.

One such resuscitation fluid that has shown promise in a mouse model of TBI + HS is a cell-free hemoglobin (Hb) solution in which the Hb is polynitroxylated and conjugated with polyethylene glycol (PEG). Resuscitation with a 4% solution of polynitroxylated PEGylated hemoglobin (PNPH, aka SanFlow™) was found to restore MAP with smaller infusion volumes than those needed for lactated Ringer’s (LR) solution or shed blood [2, 3]. It was also found to decrease cerebral edema and hippocampal neurodegeneration [4]. Conjugation of Hb with PEG serves to enlarge the molecular radius and reduce filtering in the kidney, reduce antigenicity, and increase oncotic pressure, thereby permitting it to be used as a small volume resuscitation fluid after hemorrhage [5–7]. Polynitroxylation of Hb confers superoxide dismutase [8] and catalase mimetic activity [9, 10] that reduces leukocyte adhesion to the endothelium [11, 12], limits neuronal cell death in vitro from glutamate excitotoxicity, and mitigates the neuronal toxicity associated with native Hb [4]. Furthermore, because PNPH is stored as carboxy-hemoglobin (COHb) to limit spontaneous methemoglobin (metHb) formation, PNPH acts as a carbon monoxide (CO) donor upon infusion. Low levels of CO can exert vasodilatory, anti-inflammatory, and anti-apoptotic effects [13] and may thereby provide additional mechanisms of neurovascular protection. In a model of middle cerebral artery occlusion, both PNPH [14] and PEG-COHb [15] promote dilation of collateral arteries and reduce infarct volume.

One concern with use of Hb-based oxygen carriers in humans is that humans do not synthesize ascorbic acid, which is ordinarily required for the reduction of metHb [16]. When metHb and peroxide accumulate, the result is increased formation of highly reactive ferryl Hb and oxidative damage [17]. Most animal species in which Hb-based oxygen carriers have been tested are capable of synthesizing ascorbic acid. One exception is the guinea pig [18]. Therefore, we elected to test PNPH in the guinea pig to determine if neuroprotection from TBI + HS can be sustained in a species that does not synthesize ascorbic acid. We tested the hypothesis that guinea pigs resuscitated from TBI + HS with PNPH have more remaining viable neurons in the vulnerable hippocampus, which undergoes secondary neurodegeneration, than do guinea pigs resuscitated with LR solution. Because definitive treatment with whole blood is not always available immediately in the military combat environment or in situations with large-scale civilian casualties, we elected not to add subsequent blood treatments so that we could assess whether treatment with PNPH alone is capable of sustaining neuroprotection.

## Methods

### PNPH solution

PNPH was prepared by AntiRadical Therapeutics, LLC, manufacturing facility located at Sanford Research (Sioux Falls, SD) according to an established and patented technology comprising the following steps: (i) purification of bovine red blood cells (RBCs) from platelets, white blood cells, and plasma proteins via a multistep differential centrifugation and leukocyte removal filter, (ii) extraction of Hb from purified RBCs by dialysis-ultrafiltration against a hypotonic solution under increased hydrostatic pressure, (iii) conversion of the extracted tetrameric oxyhemoglobin to COHb by exposure to CO, (iv) pasteurization of the COHb to denature and precipitate non-heme proteins and eliminate temperature-sensitive viral pathogens, (v) removal of lipid contaminants by a solid-phase extraction process, (vi) removal of heat-resistant viruses by nanofiltration through an advanced hollow fiber technology; (vii) removal of endotoxin by affinity membrane chromatography, (viii) dialysis-concentration of the COHb, (ix) PEGylation of the COHb with α-succinimidyloxysuccinyl-*ω*-methoxy polyoxyethylene, MW 5 kDa (NOF Corporation, Kanagawa, Japan), (x) polynitroxylation of PEGylated COHb with 4-(2-bromoacetamido)-2,2,6,6-tetramethyl-1-piperidinyloxy (BrAcTPO; Sigma-Aldrich, St Louis, MO) in a BrAcTPO:Hb molar ratio of 10:1, (xi) dialysis-ultrafiltration against LR, USP (B Braun, Bethlehem, PA), and (xii) 0.2 µm terminal sterilization, filling, packing, and storage at 4 °C.

The typical physicochemical characteristics of the final PNPH solution are: total Hb = 4.0 ± 0.1 g/dL; oxyhemoglobin < 5%; metHb < 5%; COHb > 95%; pH = 7.2 ± 0.2; sodium = 130 mmol/L; potassium = 4 mmol/L; chloride = 110 mmol/L; sodium lactate = 28 mmol/L; ionized calcium = 1.2 ± 0.1 mmol/L; osmolarity = 300–325 mOsm/kg; endotoxin < 0.25 EU/mL; MW = 100–110 kDa.

### Surgical preparation

All procedures on guinea pigs were approved by the Johns Hopkins University Animal Care and Use Committee and by the Animal Care and Use Review Office of the US Army Medical Research and Materiel Command (Fort Detrick, MD). We adhered to the Animal Welfare Act Regulations and other federal statutes relating to animals and experiments involving animals and the principles set forth in the current version of the *Guide for the Care and Use of Laboratory Animals*, National Research Council.

**Fig. 1:**
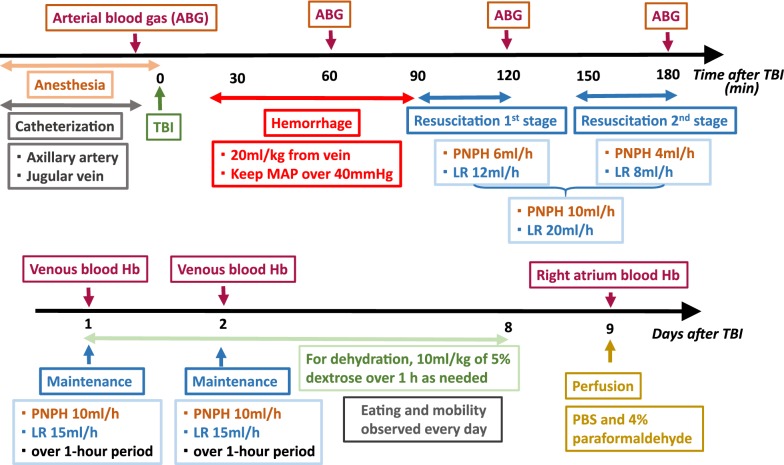
Schematic outline of time line of experimental protocol during the first 3 h of recovery and during the 9-day survival period

The experimental protocol is delineated in Fig. 1. Anesthesia of male Hartley guinea pigs (Charles River) weighing 650 ± 110 g was induced with 4% isoflurane. After a surgical plane of anesthesia was achieved, as assessed by the lack of limb withdrawal to paw pinching and by looseness of muscle tone in the jaw, anesthesia was maintained with 2% isoflurane in approximately 30% O_2_ with spontaneous ventilation. The antibiotic enrofloxacin (10 mg/kg) was injected intramuscularly before surgery and for the first 4 days of recovery. Through a 2-cm neck incision, an external jugular vein was isolated by blunt dissection. The vein was ligated with sterile suture, and a venous catheter (VAHBPU-T22; INSTECH Inc., Plymouth Meeting, PA, USA) was advanced toward the heart and secured with another ligature. For arterial catheterization, we chose the axillary artery because occlusion of the carotid artery could limit cerebral blood flow after TBI and the iliac artery gives rise to multiple small femoral branches that are difficult to cannulate. A 2-cm incision was made in the axilla, and the axillary artery was isolated by blunt dissection. The artery was ligated and a tapered arterial catheter (Solomon Tapered FunnelCath C20-10; Access Technologies Inc., Salem, OR, USA; tapered from 0.7 mm at exit to 0.4 mm outer diameter at tip) was advanced into the ascending aorta. The catheters were filled with heparinized saline, and the ends were tied off with a knot. The catheters were tunneled subcutaneously to the back of the neck, where they exited through a small incision were secured in a loop with stay sutures.

### Traumatic brain injury

Guinea pigs were injected with fentanyl (5 µg/kg) through the jugular vein catheter while maintained on isoflurane anesthesia and then placed in a stereotactic head holder. A 7 mm craniotomy was performed over the left parietal cortex centered − 4 mm from the bregma and 4 mm lateral to the midline. The dura was left intact. Once the cranial surgery was complete, the isoflurane concentration was decreased to 1% for 10 min before TBI was produced by a controlled cortical impact (CCI). The CCI device (Custom Design and Fabrication, Inc., Sandston, VA, USA) uses a linear electric motor to control an impactor with a 5 mm diameter cylinder and a beveled edge. We used a 3 mm inward displacement of the dura at a velocity of 5 m/s for a duration of 50 ms. After CCI, isoflurane and supplemental O_2_ were discontinued, and the bone flap was inserted into the hole and sealed with tissue glue. The scalp incision was closed with interrupted stitches of 4-0 silk suture. Surgical incisions were infiltrated with lidocaine hydrochloride cream (2.0%) for postoperative comfort.

### Hemorrhage and resuscitation

After the incision was closed, the guinea pig was placed in a restraint box with an open top to prevent it from turning about during hemorrhage and subsequent fluid resuscitation because the arterial catheter was connected to a pressure transducer. Normally, guinea pigs are less active in their cages than are rats and mice and often remain stationary for extended periods of time. Thus, they were able to tolerate being placed in a box that limited their mobility. If the guinea pig became very active, it was infused with an additional 5 µg/kg dose of fentanyl. Guinea pigs have lower arterial blood pressure (~ 60 mmHg) than most other mammals [18]. Because arterial pressure was further depressed by isoflurane anesthesia to 40–45 mmHg in our experiments and because anesthetics can depress baroreflexes, we waited until 20 min after CCI and the termination of isoflurane before starting the hemorrhage, at which time the guinea pigs were awakening. Hemorrhage was produced by withdrawing 20 mL/kg of blood from the jugular vein catheter. When MAP fell to 35–40 mmHg, blood withdrawal was temporarily stopped until MAP began to recover above 40 mmHg. When the targeted amount of blood had been removed, withdrawal was ceased and MAP was allowed to increase above 40 mmHg. Thus, we used a variable rate of blood withdrawal to achieve a fixed volume of hemorrhage while attempting to avoid MAP < 35 mmHg and minimize the risk of cardiogenic shock.

The guinea pigs were randomized to resuscitation with LR solution (20 mL/kg) or PNPH (10 ml/kg). These doses were based on those used in mice that underwent CCI and 20 mL/kg hemorrhage [4]. Because PEGylation conveys hyperoncotic properties to the molecule, smaller infusion volumes of PNPH are needed to restore MAP than LR. Rapid fluid resuscitation causes a rapid increase in afterload, which can lead to pulmonary edema when cardiac contractility is already compromised by low perfusion pressure. Cerebral edema can also be worsened [19]. Therefore, we performed fluid resuscitation in two 30 min stages separated by a 30 min interval to allow time for redistribution of fluid between compartments. In the first stage, 12 mL/kg LR or 6 mL/kg PNPH was infused continuously for 30 min at a constant rate beginning at 90 min from the onset of hemorrhage. The infusion was paused between 120 and 150 min. In the second stage, 8 mL/kg LR or 4 mL/kg PNPH was infused continuously for 30 min beginning at 150 min from the onset of hemorrhage. Thus, a total of 20 mL/kg of LR or 10 mL/kg of PNPH was infused between 90 and 180 min after the onset of hemorrhage. Buprenorphine (50 µg/kg) was injected subcutaneously as an analgesic at this time. An additional sham-operated group underwent catheterization and craniotomy, but no CCI or hemorrhage.

Throughout the period of hemorrhage and resuscitation, arterial blood pressure was recorded by LabChart (AD Instruments Inc., Colorado Springs, CO, USA), and arterial blood samples (0.5 mL) were obtained hourly. The samples were analyzed for pH, blood gases, Hb concentration, metHb, COHb, and glucose with an ABL800Flex (Radiometer Inc., Brea, CA USA). In the group receiving PNPH, the remaining blood sample was centrifuged to obtain plasma, which was then analyzed for Hb concentration. Rectal temperature also was monitored.

On the first and second days of recovery, a 0.5 mL blood sample was withdrawn from the jugular vein catheter for analysis of total blood Hb concentration, metHb, and COHb. The plasma Hb was also analyzed in the PNPH group. Because the tapered tip of the arterial catheter was small, it usually could not be kept patent for 24 h; therefore, arterial blood gases were not analyzed on these days. To help maintain the plasma concentration of PNPH over the first 2 days of recovery when much of the secondary injury process continues to occur, the PNPH group received additional infusions of 10 mL/kg PNPH. Similarly, the LR group received additional infusions of 15 mL/kg LR on the first and second day. These infusions occurred over a 1 h period. On the terminal day of the experiment, a blood sample was obtained from the right atrium for Hb analysis.

All groups had free access to water and food, including Timothy hay and gel food packets. They were housed in groups of 3–4 in their home cages. However, food and water intake appeared low over the first few days of recovery from TBI + HS. Both TBI + HS groups received an intravenous infusion of normal saline with 5% dextrose (10 mL/kg over a 1 h period) later in the day after receiving LR or PNPH and again on the first 2 days of recovery. Some received additional daily infusions with an amount that depended on their health condition (appetite, dehydration, weight loss, mobility). Eating and mobility were observed every day.

All guinea pigs were euthanized by first deeply anesthetizing them in a chamber with 4% isoflurane. After they became unconscious and the limbs were flaccid, the paw was pinched to ensure that the withdrawal reflex was absent. Then a sternotomy was quickly performed, an incision was made in the apex of the left ventricle, and a catheter was inserted through the incision and into the aorta. The aortic catheter was perfused with ice cold phosphate-buffered saline (PBS) followed by 300–400 mL of cold 4% paraformaldehyde while blood was allowed to drain from an incision in the right atrium.

### Neuropathology

#### Cresyl violet staining

Brains were carefully dissected and post-fixed overnight in a solution of 4% paraformaldehyde and 30% sucrose in PBS at 4 °C. Cryoprotected brains were frozen and embedded in 12% gelatin B mixed with egg yolk in a 1:1 ratio, followed by 4% paraformaldehyde and 30% sucrose. Each brain was cut on a microtome into 60-µm-thick coronal sections and serially allocated into 24 wells. Every 12th serial section was stained with cresyl violet after dehydration in a series of increasing alcohol concentrations.

#### Hippocampal viable neuron counting

We included sections from the anterior dorsal hippocampus (approximately 8.2 mm through 4.6 mm anterior to the interaural line) to estimate total neuronal survival of the CA1 + 2 and dentate gyrus pyramidal cell layers of the ipsilateral hippocampus. Stereological measurements were made by using a Nikon Eclipse 90i microscope (Nikon, Tokyo, Japan) attached to a Qimage Retiga-2000R camera, which was connected to a workstation with Stereo Investigator software (Version 10; MicroBrightField, Williston, VT, USA). Every 12th section was examined, placing the analyzed sections 720 μm apart. Using a 2 × objective, we traced the CA1 and CA2 and the dentate gyrus by outlining the regions of interest. Then we counted the viable neurons under a 40× objective. We identified viable neurons as being Nissl-positive with normal morphology and intact nucleolus and that had a nucleus that first came into focus within the optical dissector counting frame. The counting frame was 50 × 50 μm with a grid size of 300 × 300 μm. The dissector height was set at 10 μm with an upper and lower guard zone of 1 μm.

### Volumetric analysis

Histologic sections containing cortex (approximately 15.0 mm through 3.8 mm anterior to the interaural line) were used to estimate the contralateral hemisphere volume, hippocampus volume, and brain ventricular volume (excluding 3rd and 4th ventricle). After scanning, we traced the areas of the lesion, the contralateral hemisphere, the entire hippocampus, and the lateral ventricles with Image J software on every 12th of the serially cut 60 µm slices spaced 720 µm apart. Cresyl violet was used to counterstain tissue sections for histologic structure identification. After outlining the brain regions of interest, we calculated the volume by summing section area × 720 µm spacing along the entire length of the cerebral hemisphere. Lesion volume percentage was calculated by the formula: lesion volume/contralateral hemisphere volume × 100%. The observer for hippocampal neuronal counting and the volumetric measurements was blinded to treatment group.

### Statistical analysis

Guinea pigs undergoing TBI + HS were randomized to receive either LR or PNPH as a resuscitation fluid. To estimate sample size, we used the published data from the mouse model wherein the density of viable CA3 neurons increased from 12.7 in an LR group to 18.5 in a SanFlow group [4]. With an effect size of 5.8 and a standard deviation (SD) of 4.2, a sample size of 12 was estimated to provide 90% power in a two-sided test. Guinea pigs that died before perfusion for histology were replaced to obtain a final sample size of 12. We also studied a sham-operated group of 6 guinea pigs with the same surgery and survival duration and a group of 6 naïve guinea pigs with no previous surgery. The primary outcome was viable hippocampal neurons, and the secondary outcomes were lesion volume, hippocampus volume, and ventricular volume. To test for significant differences among groups at the 0.05 level, we subjected histological and volumetric data to one-way analysis of variance (ANOVA). If the *F* value was significant, individual groups were compared with the Holm-Sidak procedure for multiple comparisons. Physiologic data were analyzed among groups by two-way repeated-measures ANOVA, where treatment was a between-subject factor and time was a within-subject factor. If the *F* value for the overall effect of treatment or the interaction between treatment and time was significant, post hoc comparisons at individual time points were contrasted among groups with the Holm-Sidak procedure. If normality or equal variance tests failed, then data were subjected to the Kruskal–Wallis nonparametric test. *P* < 0.05 was considered significant in all tests. Unless otherwise noted, data are expressed as mean ± SD.

## Results

### Mortality

After TBI + HS, 17 guinea pigs were resuscitated with LR. Of these, 1 died on day 1, 1 died on day 2, and 2 died on day 9 before they could be perfused with fixative. One guinea pig was excluded from analysis because the lesion from the contusion extended into the hippocampus and interfered with the ability to perform stereology. Of the 14 guinea pigs resuscitated with PNPH, 1 died on day 4 and 1 died on day 5. The mortality rate with PNPH (2/14 = 14%) was not significantly different from that with LR (4/17 = 24%).

### Physiologic data

**Fig. 2:**
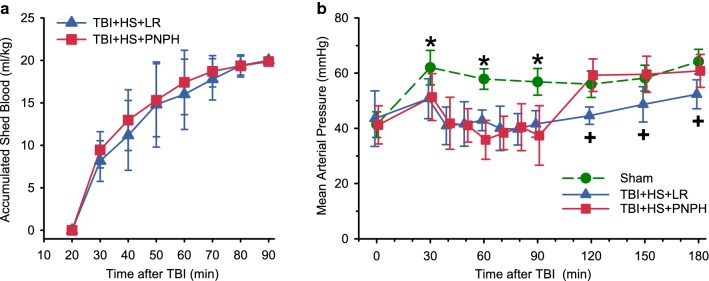
Accumulated shed blood and mean arterial blood pressure. **a** Time course of blood withdrawn after TBI for the two groups that were subsequently resuscitated with lactated Ringer’s (LR) or PNPH (mean ± SD; *n *= 12). Because blood withdrawal was stopped temporarily when mean arterial pressure declined to below 35–40 mmHg, the rate of hemorrhage differed among guinea pigs, but the average rate did not differ between the LR and PNPH groups. **b** Mean arterial pressure in guinea pigs subjected to sham operation (*n* = 6) or TBI at time 0, during hemorrhage (20–90 min), and during fluid resuscitation with either LR or PNPH (90–180 min). *P < 0.05 versus both TBI + hemorrhagic shock (HS) groups; ^+^*P* < 0.05 versus sham and LR resuscitation groups

Because hemorrhage was temporarily stopped when MAP decreased below 35–40 mmHg to avoid cardiogenic shock, the rate of hemorrhage differed among guinea pigs and, in some, was extended over an hour to achieve the goal of 20 mL/kg withdrawal. However, the accumulated amount of blood withdrawal over time did not differ between the groups that were subsequently resuscitated with LR and PNPH (Fig. 2a).

After completion of the catheterization and craniotomy, MAP was approximately 40–45 mmHg in all groups. With cessation of isoflurane anesthesia at time 0 and infusion of fentanyl, MAP increased by approximately 20 mmHg after 30 min in the sham-operated guinea pigs (Fig. 2b). This level of MAP is comparable to what others have reported in unanesthetized guinea pigs [18]. In both groups subjected to TBI and hemorrhage, the increase in MAP at 30 min was attenuated and thereafter controlled at approximately 40 mmHg until resuscitation at 90 min after TBI. Resuscitation with 20 mL/kg LR between 90 and 180 min increased MAP, but it remained less than that in the sham group (Fig. 1b). In contrast, resuscitation with 10 mL/kg PNPH between 90 and 180 min rapidly restored MAP to levels seen in the sham group and to levels greater than that obtained with LR resuscitation.

**Fig. 3:**
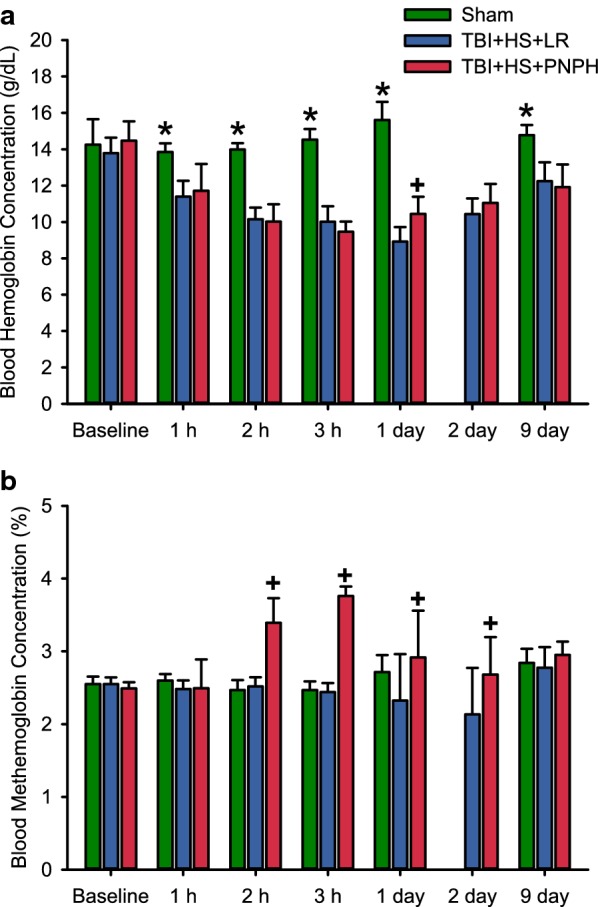
Hemorrhage produces the expected decreases in whole blood hemoglobin (**a**), whereas PNPH infusion produces small increases in whole blood methemoglobin concentration (**b**) with no immediate effect on whole blood hemoglobin concentration. Data are shown for the sham group (*n* = 6) and groups subjected to TBI and hemorrhage followed by resuscitation at 90 min with lactated Ringer’s solution (LR; *n* = 12) or PNPH (*n* = 12). *P < 0.05 versus both TBI + hemorrhagic shock (HS) groups; ^+^*P* < 0.05 versus sham and LR resuscitation groups

**Fig. 4:**
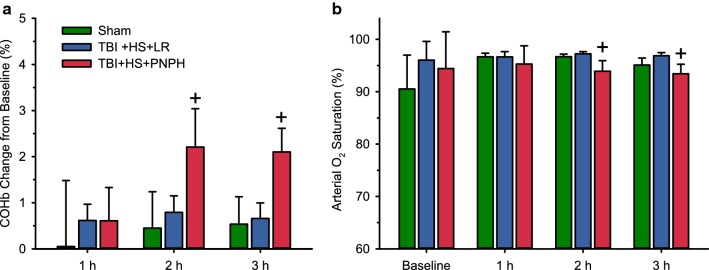
Increases in whole blood carboxyhemoglobin (**a**) after PNPH infusion had only a small negative effect on arterial O_2_ saturation of hemoglobin (**b**). Data shown for the sham group (*n* = 6) and groups subjected to TBI and hemorrhage followed by resuscitation at 90 min with lactated Ringer’s solution (LR; *n* = 12) or PNPH (*n* = 12). ^+^*P* < 0.05 versus sham and LR resuscitation groups

Total blood Hb concentration was decreased to a similar extent by hemorrhage in the LR and PNPH groups (Fig. 3A). After transfusion, Hb concentration remained similar in the two groups, although on day 1, the concentration was 1.5 g/dL higher in the PNPH group before the supplemental infusion of PNPH. Plasma Hb concentration increased from 0.02 ± 0.02 to 0.57 ± 0.08 g/dL after the infusion and declined to 0.27 ± 0.04 and 0.33 ± 0.11 g/dL on days 1 and 2, respectively, before the daily supplemental infusions were started on these days. By 9 days, the plasma Hb concentration returned to baseline. The stock solution of PNPH contained 5% metHb at the time of infusions. The infusion of PNPH resulted in a 1.3% increase in metHb (percent of total Hb) at the end of the infusion and a 0.7% increase on days 1 and 2 before the additional infusions (Fig. 3b). Furthermore, PNPH has bound CO to limit metHb formation during storage. After infusion, CO is released from PNPH and equilibrates with erythrocyte Hb. After infusion of PNPH, total blood COHb was found to increase by only 1.6% of total Hb above the 1-h post-TBI value (Fig. 4a). The arterial O_2_ saturation declined accordingly but remained greater than 90% (Fig. 4b).

At the end of surgery, these anesthetized guinea pigs with spontaneous ventilation exhibited hypercapnia and acidosis, but tend to normalize after discontinuing anesthesia after TBI (Table 1). Arterial pH, PCO_2_, and glucose concentration were comparable between the TBI groups during hemorrhage and reperfusion, although pH was somewhat lower in the sham group. Arterial PO_2_ was lower at the end of surgery in the sham group because supplemental O_2_ was discontinued at this time. The total volume of supplemental 5% dextrose in normal saline infused on days 1 and 2 was similar in the LR group (28.5 ± 4.2 mL) and PNPH group (30.8 ± 4.1 mL) as per protocol. Subsequent infusions were based on the health of the animal. The sum of infused 5% dextrose in normal saline from day 3 to day 8 was not significantly different between the LR group (39.7 ± 5.1 mL) and PNPH group (36.9 ± 5.5 mL). This implies that the trajectory for their overall health recovery (appetite, hydration status, mobility) did not differ markedly by treatment.

**Table 1:** Arterial pH, blood gases, and blood glucose concentration of guinea pigs

Parameter	End surgery	1 h	2 h	3 h
pH				
Sham	7.18 ± 0.07	7.33 ± 0.03*	7.32 ± 0.04*	7.30 ± 0.02*
TBI + HS + LR	7.20 ± 0.05	7.41 ± 0.04	7.43 ± 0.07	7.39 ± 0.03
TBI + HS + PNPH	7.21 ± 0.05	7.39 ± 0.02	7.42 ± 0.04	7.40 ± 0.04
PCO_2_ (mmHg)				
Sham	42 ± 4	32 ± 2	30 ± 3	32 ± 3
TBI + HS + LR	45 ± 8	30 ± 3	28 ± 6	30 ± 3
TBI + HS + PNPH	45 ± 7	31 ± 4	32 ± 4	32 ± 2
PO_2_ (mmHg)				
Sham	79 ± 19*	109 ± 8	106 ± 7	89 ± 11
TBI + HS + LR	159 ± 68	108 ± 16	116 ± 13	108 ± 15
TBI + HS + PNPH	188 ± 104	109 ± 27	126 ± 22	111 ± 24
Glucose (mg/dL)				
Sham	145 ± 148	86 ± 34	77 ± 18	84 ± 20
TBI + HS + LR	149 ± 107	111 ± 43	105 ± 24	108 ± 30
TBI + HS + PNPH	162 ± 46	114 ± 47	96 ± 42	90 ± 29

*HS* hemorrhagic shock, *LR* lactated Ringer’s solution, *PCO*_*2*_ partial pressure of carbon dioxide, *PNPH* polynitroxylated PEGylated hemoglobin, *PO*_*2*_ partial pressure of oxygen, *TBI* traumatic brain injury. *P < 0.05 versus TBI + HS + LR and versus TBI + HS + PNPH

### Neuropathologic data

**Fig. 5:**
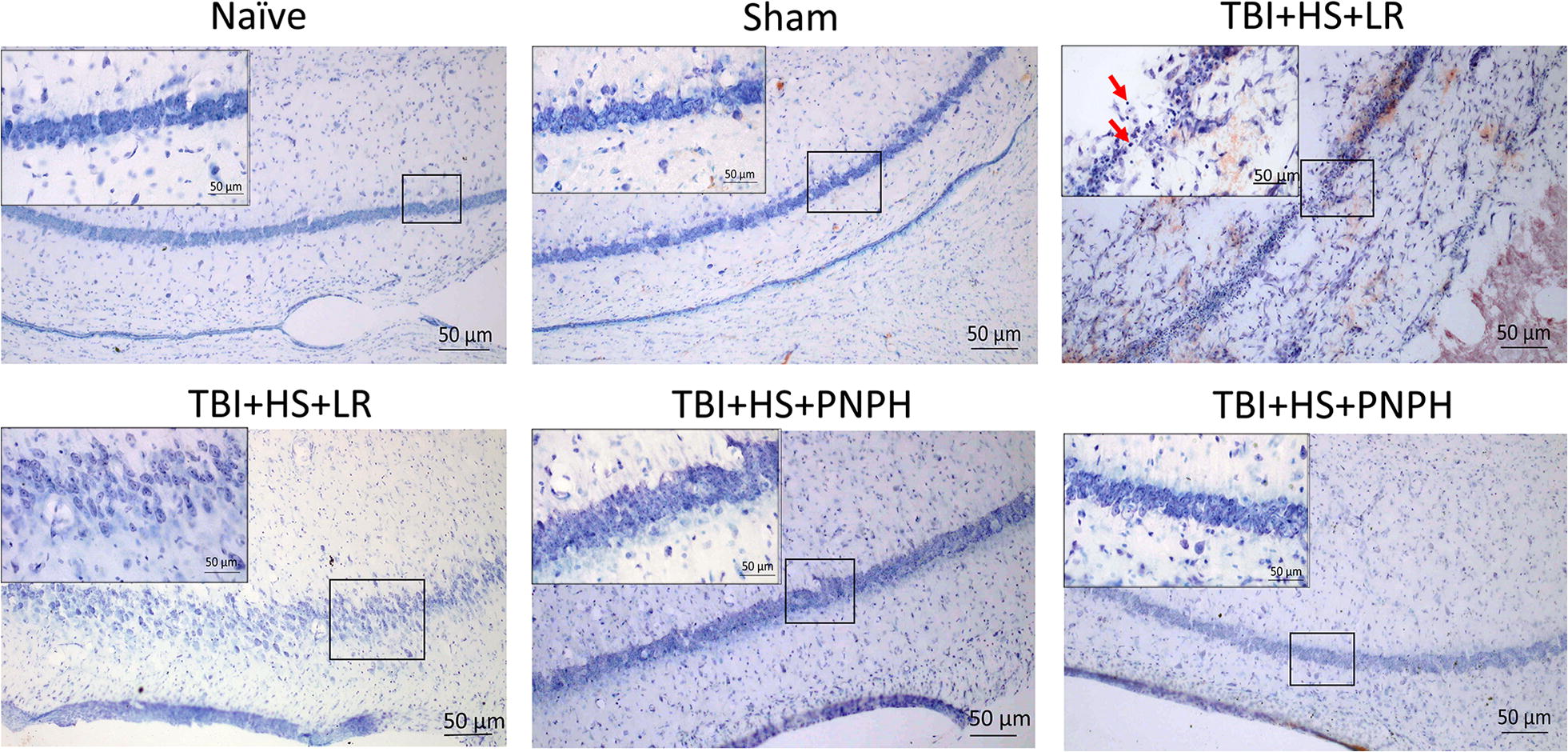
Representative cresyl violet-stained sections of CA1 + 2 hippocampus from a naïve guinea pig with no surgery, a sham-operated guinea pig, and guinea pigs subjected to TBI and hemorrhagic shock (HS) followed by resuscitation with lactated Ringer’s solution (LR) or PNPH. Naïve and sham: Nissl-positive cells in the naïve group and sham group had normal morphology and intact nucleolus, indicating viable neurons. TBI + HS + LR: CA1 + 2 region had areas of cell loss accompanied by neutrophil infiltration. In some cases, a loose arrangement of cell bodies was observed, though the morphology of individual neurons looked normal. Red arrows show dead neurons with dark staining. TBI + HS + PNPH: Neurons in the CA1 + 2 region usually showed the normal compact arrangement. Insets in each image show higher magnification of boxed area. Scale bars = 50 μm

**Fig. 6:**
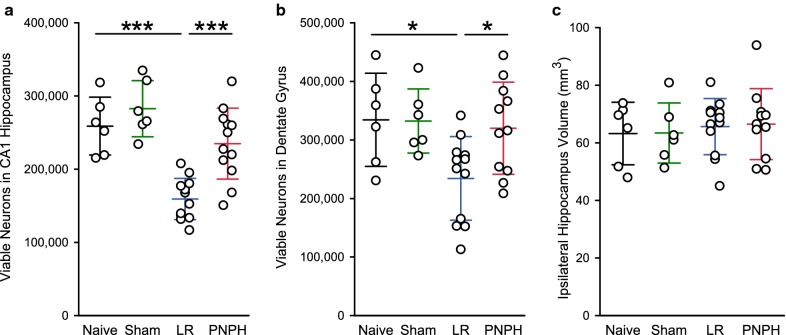
Comparisons of viable neuron numbers and hippocampal volume. Unbiased stereology estimate of total number of viable neurons in CA1 + 2 hippocampus (**a**), dentate gyrus (**b**), and volume of entire hippocampus (**c**) in the naïve group (*n* = 6), the sham group (*n* = 6), and groups subjected to TBI and hemorrhage followed by resuscitation at 90 min with lactated Ringer’s solution (LR; *n* = 12) or PNPH (*n* = 12). Mean ± SD and individual data for each guinea pig are shown. ***P < 0.001 versus TBI + hemorrhagic shock group resuscitated with LR; *P < 0.05 versus TBI + hemorrhagic shock group resuscitated with LR

Areas of selective neuronal loss were evident in hippocampus after TBI + HS in the LR group but not in the naïve and sham-operated groups (Fig. 5). In the PNPH group, these areas of neuronal loss were less prominent. Quantification of viable neurons across the entire length of the CA1 + 2 and dentate gyrus regions with unbiased stereology did not reveal significant differences between the naïve and sham groups (Fig. 6a, b). However, the number of viable neurons was decreased in the LR-treated TBI + HS group in CA1 + 2 (*P* < 0.001) and in dentate gyrus (*P* < 0.05), whereas the corresponding numbers in the PNPH-treated TBI + HS group did not differ significantly from the values in the naïve and sham groups. Compared to LR treatment, PNPH treatment significantly rescued the number of viable neurons in CA1 + 2 from 59 ± 10% (% of the naive + sham mean value) to 87 ± 18% (*P* < 0.001) and in dentate gyrus from 70 ± 21% to 96 ± 24% (*P* < 0.05). The volume of the entire hippocampus, which was measured on serial sections, was similar in all groups, consistent with the lack of infarction or direct mechanical injury to the hippocampus (Fig. 6c).

**Fig. 7:**
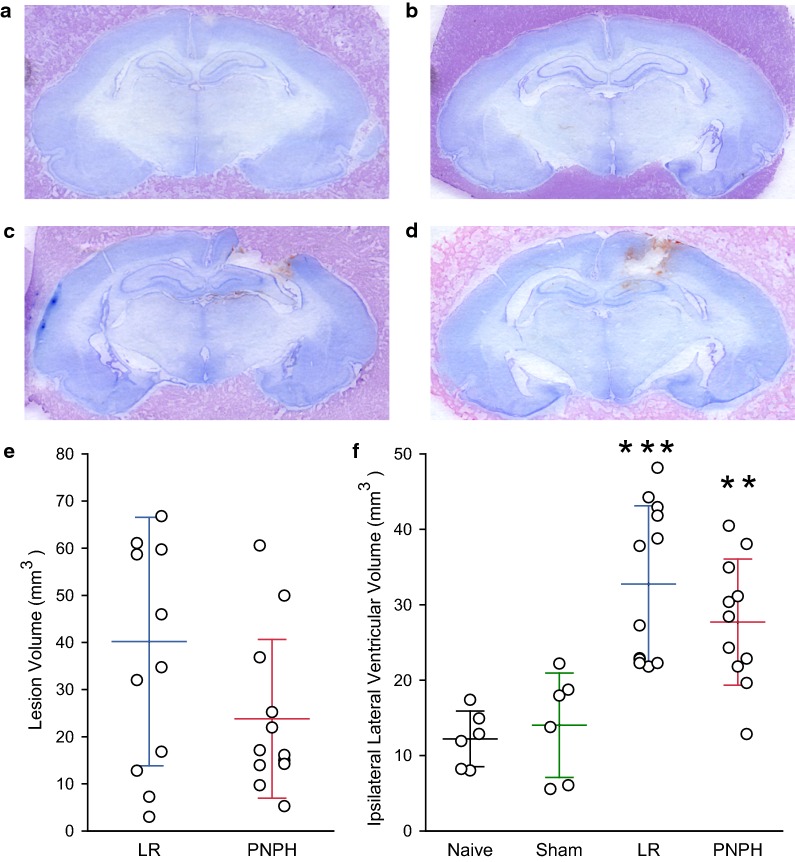
Effect of PNPH on lesion volume. Representative cresyl violet-stained images of coronal brain sections surrounded by embedding material from a naïve guinea pig with no surgery (**a**), a sham-operated guinea pig (**b**), and guinea pigs subjected to TBI and hemorrhagic shock (HS) followed by resuscitation with lactated Ringer’s (LR) solution (**c**) or PNPH (**d**). By 9 days of recovery from TBI, cavitation and areas of poor tissue staining typically extended through much of the cortical layers and into white matter, with sparing of the hippocampus. E, Lesion volumes of guinea pigs subjected to TBI and HS followed by resuscitation at 90 min with lactated Ringer’s solution (LR; *n* = 12) or PNPH (*n* = 12). F, Volume of the ipsilateral lateral ventricle in the naïve group (*n* = 6), the sham group (*n* = 6), and groups subjected to TBI and HS followed by resuscitation with LR or PNPH. Mean ± SD and individual data for each guinea pig are shown. **P < 0.01, ***P < 0.001 versus both naïve and sham groups

Representative cresyl violet-stained images of coronal brain sections are shown from a naïve (Fig. 7a), sham-operated (Fig. 7b), TBI + HS guinea pigs with LR (Fig. 7c) and PNPH (Fig. 7d) resuscitation. At 9 days of recovery, cavitation with some remnants of hemorrhage are evident in the cerebral cortex and underlying white matter. Lesion area measurements included the cavity and tissue with sparse cresyl violet staining. PNPH administration produced a non-significant decrease in lesion volume (*P* = 0.083, 2-tailed *t* test) compared to LR administration (Fig. 7e). The volume of the lateral ventricle on the impacted side was also assessed on serial sections of the fixed tissue. As expected, significant ventricular enlargement was detected after TBI (*P* < 0.001), in some cases increasing in volume up to threefold (Fig. 7f). This enlargement was 23% less in the PNPH-infused group than in the LR-infused group, but the difference did not attain statistical significance.

## Discussion

The main finding of this study is that resuscitation from TBI + HS with PNPH substantially reduced neurodegeneration in the CA1 + 2 and the dentate gyrus regions of hippocampus of guinea pigs. This improvement was associated with a more rapid restoration of MAP despite the infusion volume being smaller than that for LR. Because PNPH infusion over the first 2 days without the use of additional whole blood infusions attenuated delayed neurodegeneration at 9 days, these results infer that PNPH may be of use in situations where definitive treatment is delayed [1].

We observed secondary neuronal cell loss in CA1 + 2 and dentate gyrus regions of the hippocampus beneath the primary contusion in this model. The cell loss occurred without a measurable change in overall volume of the hippocampus and without overt infarction. This type of injury is consistent with selective neuronal cell death attributable to excitotoxicity and oxidative stress. Work by others in primary neuronal culture showed that addition of PNPH to the media did not induce cell death, in contrast to the toxicity of native Hb or PEG-Hb without nitroxide moieties [4]. Importantly, PNPH decreased cell death induced by stretch or exposure to glutamate and glycine. Thus, PNPH has the ability to directly protect neurons.

Although the average lesion volume in cerebral cortex was 41% smaller in the PNPH-resuscitated group, the variability was relatively large and the difference from the LR-resuscitated group did not attain statistical significance. The observed ventriculomegaly in perfusion-fixed brain in both TBI + HS groups presumably reflects tissue loss in the entire hemisphere [20] and may include tissue loss beyond the primary lesion in the cerebral cortex. This measurement had less relative variability than did lesion volume. Although the increase in volume of the lateral ventricle ipsilateral to the TBI was 23% less in the PNPH group than in the LR group on average, this attenuation also did not attain statistical significance. Thus, the study did not have sufficient power with a sample size of 12 to detect effects of these secondary measurements with two-sided tests.

Resuscitation with PNPH restored MAP more rapidly and effectively than resuscitation with LR despite the infused volume of PNPH being half that of shed blood. This rapid restoration is most likely attributable to the oncotic effect of the PEG moieties on PNPH and has also been seen by others in a mouse model of TBI + HS [2]. Furthermore, the increase in brain water content was attenuated by PNPH treatment and, consequently, cerebral perfusion pressure was reported to be improved in this mouse model [2]. Although we did not measure intracranial pressure or cerebral blood flow in the present study, it should be noted that transfusion of PNPH does not cause pial arteriolar constriction in uninjured rats or in the context of focal cerebral ischemia [14].

In a similar model in mice with CCI followed by 90 min of hemorrhagic hypotension, resuscitation with PNPH provided neuroprotection in CA3 hippocampus [4]. The experimental protocol we used in guinea pigs differed in some respects in that the guinea pigs were unanesthetized during the hemorrhage and resuscitation and they received additional PNPH infusions and blood sampling on the first and second days after resuscitation, whereas the mice were transfused with shed blood 1 h after the PNPN infusion (2.5 h after TBI) to simulate early definitive care. In addition, we conducted a complete, unbiased stereologic analysis of viable cell counts throughout the entire CA1 + 2 and dentate gyrus regions of the hippocampus. Typically, guinea pigs are not used in TBI models, and we are not aware of previous studies that have employed the CCI model in them. One challenge in using guinea pigs is that the femoral arteries are difficult to cannulate because they divide into multiple small branches. Most studies of hemodynamics in guinea pigs use carotid artery catheterization [18, 21], but we avoided this method because it would likely impair cerebral blood flow under conditions of TBI + HS. Hence, we performed catheterization of an axillary artery. However, catheterization of this artery also is challenging because of its small size, requiring a 0.4-mm outer diameter tapered catheter. Guinea pig arteries tend to be more fragile with less connective tissue, possibly because their arterial blood pressure is relatively low compared to that in other rodents.

MetHb can promote oxidative injury and thereby counteract the neuroprotective properties of PNPH. The primary reason that we replicated the study in guinea pigs is their inability to synthesize ascorbic acid, which is used in the reduction of metHb. Because humans also are unable to synthesize ascorbic acid, evaluation of PNPH efficacy in guinea pigs is important for appraising its potential translatability to clinical use. The stock solution of PNPH had approximately 5% metHb at the time of use. After infusion of 10 mL/kg PNPH, the metHb concentration in whole blood increased by 1.3% above baseline and then declined to a 0.6% increase relative to the LR group by the next day. These levels are not excessive and are less than half of the 4.6% increase in metHb obtained after infusion of twice the PNPH dose (20 mL/kg) in mice [2]. Thus, the guinea pig does not appear to be more prone to forming metHb after PNPH infusion. This observation is in contrast to that observed with transfusion of an early generation of polymerized Hb wherein metHb exposure was fivefold greater in the guinea pig than in the rat [18]. The selective lack of PNPH-induced augmentation of metHb in guinea pigs is likely attributable to the peroxidase-like activity of the nitroxides, which will limit local H_2_O_2_ availability for promoting the ferrous to ferric transition [10].

The formation of metHb in the stock solution was limited by storage in the carboxy state. After infusion of 10 mL/kg PNPH, COHb increased by approximately 1.6% in whole blood. The CO on the PNPH rapidly exchanges with the RBC-based Hb pool, which is much larger than the cell-free Hb pool, and with other heme moieties in the tissue. Consequently, the increase in COHb in whole blood has only a minor effect on overall O_2_ carrying capacity. Moreover, CO donors are known to exert anti-apoptotic, anti-inflammatory, and vasodilatory effects [13], all of which may provide neuroprotection.

The main limitation of this study is that we did not measure biomarkers of oxidative stress and did not make a comparison with a PEG-Hb solution without the superoxide dismutase/catalase activity. However, this comparison has been reported in mouse neurons where PNPH was more potent than PEG-Hb in protecting neurons from excitotoxicity [4]. The main reason this was not done in the guinea pig model was that our purpose was to demonstrate safety and to show that neuroprotection was not lost in a species that does not synthesize ascorbic acid.

## Conclusion

This study provides important safety and efficacy data of PNPH, including measurement of methemoglobin and COHb levels, as a new generation of hemoglobin-based oxygen carriers in a newly developed guinea pig model of TBI. Our results support the ability of PNPH infusion to protect the brain from secondary neurodegeneration when used as a resuscitation fluid after a combined insult of cortical impact and hemorrhagic shock. Despite the inability of the guinea pig to synthesize the ascorbic acid used in the reduction of metHb, metHb did not accumulate to excessive levels after the infusion of PNPH, suggesting that any increase in heme oxidation was balanced by heme reduction. Any augmented damage caused by the moderate increase in metHb was more than offset by other protective properties of PNPH. Thus, PNPH may serve as a first line of defense against TBI + HS for sustaining brain viability in the field or in the emergency room until advanced intensive care can be provided.

## Data Availability

Original data will be provided upon a reasonable written request.

## Abbreviations

ANOVA: Analysis of variance
CCI: Controlled cortical impact
COHb: Carboxy hemoglobin
Hb: Hemoglobin
HS: Hemorrhagic shock
LR: Lactated Ringer’s
MAP: Mean arterial pressure
PBS: Phosphate-buffered saline
PEG: Polyethylene glycol
PNPH: Polynitroxylated PEGylated hemoglobin
RBC: Red blood cell
TBI: Traumatic brain injury
